# *Pkd1* and *Pkd2* Are Required for Normal Placental Development

**DOI:** 10.1371/journal.pone.0012821

**Published:** 2010-09-16

**Authors:** Miguel A. Garcia-Gonzalez, Patricia Outeda, Qin Zhou, Fang Zhou, Luis F. Menezes, Feng Qian, David L. Huso, Gregory G. Germino, Klaus B. Piontek, Terry Watnick

**Affiliations:** 1 Division of Nephrology, Department of Medicine, Johns Hopkins University School of Medicine, Baltimore, Maryland, United States of America; 2 Department of Molecular and Comparative Pathobiology, Johns Hopkins University School of Medicine, Baltimore, Maryland, United States of America; University of Dayton, United States of America

## Abstract

**Background:**

Autosomal dominant polycystic kidney disease (ADPKD) is a common cause of inherited renal failure that results from mutations in *PKD1* and *PKD2*. The disorder is characterized by focal cyst formation that involves somatic mutation of the wild type allele in a large fraction of cysts. Consistent with a two-hit mechanism, mice that are homozygous for inactivating mutations of either *Pkd1* or *Pkd2* develop cystic kidneys, edema and hemorrhage and typically die in midgestation. Cystic kidney disease is unlikely to be the cause of fetal loss since renal function is not required to complete gestation. One hypothesis is that embryonic demise is due to leaky vessels or cardiac pathology.

**Methodology/Principal Findings:**

In these studies we used a series of genetically modified *Pkd1* and *Pkd2* murine models to investigate the cause of embryonic lethality in mutant embryos. Since placental defects are a frequent cause of fetal loss, we conducted histopathologic analyses of placentas from *Pkd1* null mice and detected abnormalities of the labyrinth layer beginning at E12.5. We performed placental rescue experiments using tetraploid aggregation and conditional inactivation of *Pkd1* with the Meox2 Cre recombinase. We found that both strategies improved the viability of *Pkd1* null embryos. Selective inactivation of *Pkd1* and *Pkd2* in endothelial cells resulted in polyhydramnios and abnormalities similar to those observed in *Pkd1^−/−^* placentas. However, endothelial cell specific deletion of *Pkd1* or *Pkd2* did not yield the dramatic vascular phenotypes observed in null animals.

**Conclusions/Significance:**

Placental abnormalities contribute to the fetal demise of *Pkd^−/−^* embryos. Endothelial cell specific deletion of *Pkd1* or *Pkd2* recapitulates a subset of findings seen in *Pkd* null animals. Our studies reveal a complex role for polycystins in maintaining vascular integrity.

## Introduction

Autosomal dominant polycystic kidney disease (ADPKD) is a common cause of inherited renal failure [1]. Affected individuals develop progressive renal cyst formation and enlargement that ultimately destroys normal kidney architecture. Mutations in *PKD1* are responsible for 85% of all cases while mutations in *PKD2* account for the remainder [2]. Both forms of the disease are associated with a similar array of extra renal manifestations, including liver cysts and cardiovascular complications such as intracranial aneurysms and mitral valve prolapse [3].

The protein products of *PKD1* (Polcystin-1, PC1) and *PKD2* (Polycystin-2, PC2) are thought to function in a common signaling pathway that has yet to be completely elucidated [4], [5]. PC1 is predicted to be a large, non-kinase membrane receptor with a multi-component extracellular N-terminus, 11 transmembrane domains and an intracellular C-terminus that is critical for signal transduction. PC2 is a Ca^2+^-permeable, TRP-like channel that interacts with the C-terminus of PC-1 forming a receptor channel complex that has been hypothesized to mediate mechanosensory signal transduction by primary cilia in several cell types including renal epithelial cells and endothelial cells [6], [7], [8].

Although both PC1 and PC2 are expressed in nearly every tissue that has been studied, the manifestations of gene loss in humans are relatively restricted and focal in nature. For example, in the kidney, microdissection studies of early stage human PKD kidneys demonstrate that cyst formation is a localized process that affects only a subset of cells in the renal tubules [9]. A two hit model of disease is consistent with this observation and is supported by genetic analyses of cystic tissues where somatic mutations affecting the wild type allele can be detected in a large fraction of cysts [10], [11].

Studies of mice with targeted mutations of *Pkd1* and *Pkd2* are in keeping with this model [10], [12], [13]. *Pkd1* and *Pkd2* heterozygous mice present with few if any renal cysts but develop dramatic cystic disease when the other allele undergoes somatic deletion at a high rate [14], [15], [16], [17]. In addition, germline homozygous mutants develop severely cystic kidneys and die *in utero* at mid-gestation [14], [15], [16], [18], [19], [20], [21], [22], [23]. Polyhydramnios, severe edema and hemorrhage are universal findings in homozygous mutants of either locus by E12.5.

Taken together, the data from mouse models suggests that ADPKD is recessive at the molecular level and that embryonic loss of either *Pkd1* or *Pkd2* is incompatible with viability. Interestingly despite the high incidence of *PKD* mutations, there have been no reports of live-born humans with inactivating, biallelic *PKD1* or *PKD2* mutations. In the only example reported in the literature, a marriage between affected first-degree cousins with *PKD1*-linked disease resulted in a higher than expected rate of fetal loss [24]. The authors concluded that biallelic mutation of *PKD1* was the likely cause of embryonic lethality.

Why does polycystin loss result in fetal death? It is unlikely that cystic renal disease is the cause of intrauterine demise since renal function is not required to complete gestation. The uniform presence of vascular fragility and edema in *Pkd* null mice along with reports of cardiac defects in some models has led to the hypothesis that the demise of these embryos is primarily cardiovascular, due to a combination of leaky vasculature and possibly cardiac defects [18], [20], [21], [22], [23], [25]. This is in keeping with the expression of polycystins throughout the embryonic and adult cardiovascular systems as well as in both endothelial cells and vascular smooth muscle cells [7], [8], [18], [26].

In this study we address the cause of lethality in *Pkd* null embryos. We show that polycystins are expressed in the murine placenta and that placental abnormalities are a likely cause of fetal demise in polycystin null animals. We also used floxed alleles of *Pkd1* and *Pkd2* to investigate the roles of polycystins in various placental cells types. Tetraploid aggregation as well as conditional knock out experiments with both Meox2 and Tie2-Cre recombinases suggest that *Pkd1* and *Pkd2* play a functional role in both trophoblast and endothelial cell compartments of the placenta. Surprisingly, endothelial loss of *Pkd1* or *Pkd2* induced by a Tie2-Cre recombinase does not recapitulate the vascular collapse observed in null animals.

## Results

### *Pkd1* and *Pkd2* are expressed in Murine Placenta

**Figure 1:**
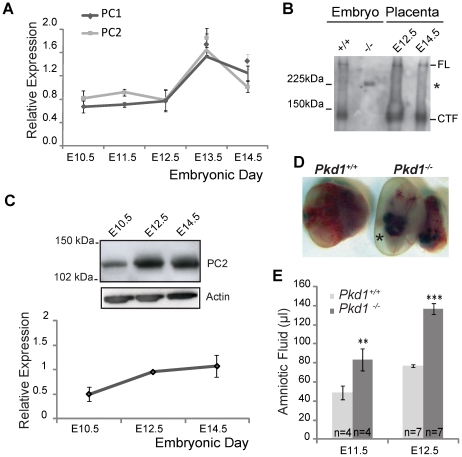
Coordinate Expression of PC1 and PC2 in Murine Placentas. A. Expression of *Pkd1* and *Pkd2* in E10.5–E14.5 placentas assayed by quantitative RT-PCR, normalized to RPL13. N = 4 placentas were analyzed at E10.5 and E14.5 and N = 3 placentas at the other time points. B. Immunoprecipitation of PC1 from placentas harvested at E12.5 and E14.5 and E12.5 embryos using a chicken antibody directed at the PC1 C-terminus. PC1 was detected on Western by rabbit antisera also directed at the PC1 C-terminus. Full length PC1 (FL) and the PC1 C-terminal cleavage fragment (CTF) are absent in the *Pkd1^−/−^* embryo. The asterisk denotes a non-specific band previously described [46]. *Pkd1^−/−^* placentas were not used as a negative control since they would be expected to contain maternal PC1. C. Expression of PC2 by Western analysis in E10.5–E14.5 placentas. Expression level was normalized to actin. N = 4 placentas analyzed at each time point. Inset shows a representative Western blot. D. Representative *Pkd1^−/−^* and littermate control (*Pkd1^+/+^*) embryos harvested at E11.5. Asterisk indicates polyhydramnios. E. Quantification of amniotic fluid. Increased amniotic fluid can be detected in *Pkd1^−/−^* embryos as early as E11.5. ** p<.01, *** p<.001.

We used RT-PCR to document the expression of *Pkd1* and *Pkd2* in murine placentas between E10.5 and E14.5 (Figure 1A). Placental expression of PC1 and PC2 could also be detected by Western analysis by E12.5 (Figure 1 B and C).

### Characterization of *Pkd1^−/−^* Null Placentas

**Figure 2:**
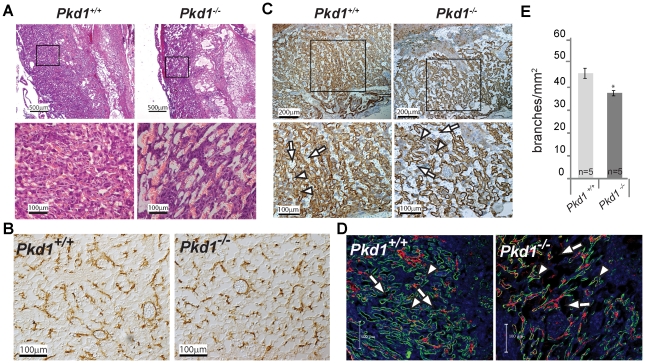
Abnormal labyrinth layer in *Pkd1^−/−^* placentas. A. Low power (upper panels) and high power (lower panels) views of congenic C57BL/6, E12.5 placentas stained with haematoxylin-eosin (H&E). B. Pericytes in E12.5 placentas stained with antibodies to alpha smooth actin (α-SMA). The density of pericytes in *Pkd1^−/−^* placentas is reduced, consistent with a decrease in the density of fetal vessels. C. Low power (upper panels) and high power (lower panels) views of E12.5 placentas stained with isolectin B4 (IB4), highlighting the matrix surrounding fetal vessels. Arrowheads denote fetal vessels. Arrows indicate maternal vascular spaces. The null placenta has fewer fetal vessels and dilated maternal spaces. D. Fetal vessels in *Pkd1^+/+^* and *Pkd1^−/−^* 12.5 dpc placenta stained with fluorescent-labeled isolectin B4 (green) and laminin (red). Nuclei stained with DAPI are blue. White arrowheads indicate fetal vessels that are outlined by IB4 and laminin. Arrows indicate maternal vascular spaces. There is a decrease in the number of fetal vessels. E. Vessel density is decreased in the labyrinth layer of *Pkd1^−/−^* placentas compared with *Pkd1^+/+^* controls (37.6±1.1 branches/mm^2^ vs. 46.3±2.3 respectively) * p<.05.

Defects in the structure and/or function of the chorioallantoic placenta are recognized to be common causes of mid-to late gestational loss of pregnancy. Since *Pkd1* and *Pkd2* were expressed in placental tissue we considered the possibility that placental failure might be the cause of fetal demise in *Pkd* null embryos [27], [28]. We systematically examined the placentas of *Pkd1^+/+^, Pkd1^+/−^* and *Pkd1^−/−^* mice at gestational ages E10.5 through E15.5 in both mixed (129Sv/BL/6) and congenic C57BL/6 backgrounds. At E10.5–E11.5, the deciduas basalis and spongiotrophoblast layers of all three genotypes were similar though the labyrinth layer of *Pkd1^−/−^* placentas appeared slightly less developed with polyhydramnios apparent as early as E11.5 (Figure 1 D and E). At later time points, the polyhydramnios became more severe and abnormalities in *Pkd1^−/−^* placentas became progressively more noticeable. In Figure 2 null placentas harvested from C57BL/6 congenic E12.5 embryos were stained for isolectin B4 (IB4), a marker that highlights the extracellular matrix surrounding fetal blood vessels [29]. We observed disorganized fetal arteriole and capillary networks and *Pkd1^−/−^* placentas exhibited a statistically significant decrease in the number of vascular branches when compared with wild type controls (Figure 2 A, C, D and E). The trophoblast lined maternal vascular channels also appeared dilated (Figure 2A, arrows in Figure 2 C and D). Small areas of separation, hemorrhage and necrosis with fibrin deposition were occasionally observed at the maternal-fetal interface. In addition, antibodies to alpha smooth actin (α-SMA) detected fewer pericytes, possibly reflecting a decrease in the number of fetal vessels (Figure 2B). Despite a decrease in vascular complexity (Figure 2E), at E12.5, there was no significant difference in either the total placental area (*Pkd1^−/−^*: 5.57±0.42 mm^2^ vs. *Pkd1^+/+^*: 6.22±0.27 mm^2^, P = .24) or in the labyrinth area between *Pkd1^−/−^* and controls. (*Pkd1^−/−^*: 2.85±.27 vs. *Pkd1^+/+^*: 3.4±.24, P = .16).

There was variability in the onset of severe placental defects, with congenic C57BL/6 *Pkd1^−/−^* mice generally exhibiting more severe abnormalities at earlier time points compared with the mixed background. This is consistent with the observation that congenic C57BL/6 *Pkd1^−/−^* embryos also die at earlier time points. *Pkd2* null placentas exhibit similar histologic abnormalities [30].

### Placental Rescue Experiments

**Figure 3:**
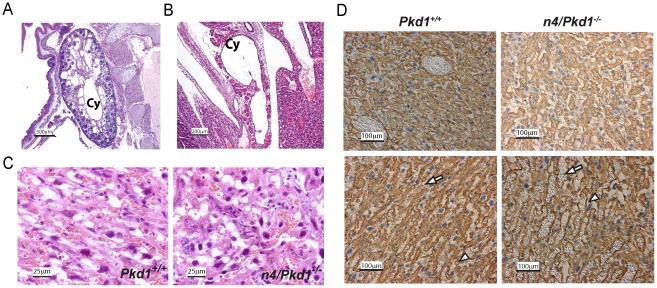
Placental Rescue by Tetraploid Aggregation. A, B. Haematoxylin-eosin stained sections of E17.5 tetraploid rescue n4/*Pkd1^−/−^* embryos show classic *Pkd1* null phenotype with renal cysts (Panel A) and pancreatic cysts (Panel B). This is because tetraploid wild type cells are not incorporated into the fetus. “Cy” denotes cysts. C. Haematoxylin-eosin stained placental sections harvested from viable E17.5 wild type (*Pkd1^+/+^*) or *Pkd1* mutant (n4/*Pkd1^−/−^*) embryos. The defect in the labyrinth layer appears improved in this placenta. D. IB4 staining of representative sections from E17.5 *Pkd1^+/+^* (left panels) and n4/*Pkd1^−/−^* placentas (right panels). There was variable improvement in the placental phenotype with the n4/*Pkd1^−/−^* placenta in the upper panel appearing to be more like the wild type placenta.

In order to explore whether a placental defect might be responsible for intrauterine demise, we performed placental rescue experiments using two strategies. First, we aggregated wild type tetraploid embryos with diploid embryos derived from *Pkd1^+/−^* intercrosses [31]. In this method, tetraploid, wild type cells contribute preferentially to extraembryonic placental tissues including trophoblasts while the embryo is derived exclusively from potentially mutant diploid cells. Our results were consistent with a partial rescue of lethality since 3/7 embryos harvested at E17.5 were viable *Pkd1^−/−^*, which is remarkable since *Pkd1* null embryos rarely survive past E15.5 (Table S1). Of the remaining embryos, three were *Pkd1^+/−^* and one was *Pkd1^+/+^*. Consistent with the enhanced viability of the “rescued” *Pkd1^−/−^* null embryos, we observed variable improvement in the placental histology (Figure 3 C and D). As expected *Pkd1^−/−^* fetuses had severe cystic kidney disease, edema and polyhydramnios due to the lack of fetal incorporation of tetraploid cells (Figure 3 A and B).

**Figure 4:**
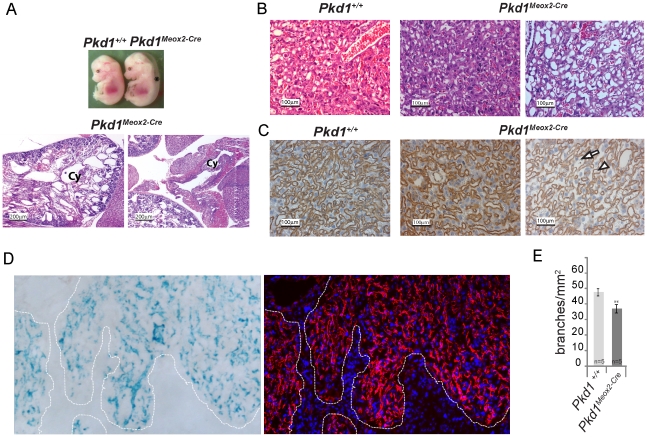
Conditional Inactivation of *Pkd1* using Meox2 Cre recombinase. A. Conditional inactivation of *Pkd1* using a Meox-2 Cre recombinase expressing mouse line results in the typical polycystin null phenotype, reflecting high activity of the recombinase in the embryo. The top panel shows the gross appearance of *Pkd1^−/cond^; Meox2-Cre^+^* (*Pkd1^Meox2-Cre^*) and *Pkd1^+/cond^; Meox2-Cre^−^* (*Pkd1^+/+^*) neonates (top panel). *Pkd1^Meox2-Cre^* mutants are severely edematous (*). The bottom panels show haematoxylin-eosin stained sections of cystic kidney (left) and cystic pancreas (right). “Cy” denotes cysts. B, C. Sections of E14.5 placentas from *Pkd1^cond/+^, Meox2-Cre^−^* (*Pkd1^+/+^*) or *Pkd1^cond/−^, Meox2-Cre^+^*(*Pkd1^Meox2-Cre^*) embryos stained with haematoxylin-eosin (B) or IB4 (C). Two representative examples of *Pkd1^Meox2-Cre^* placentas show different degrees of rescue when compared with a *Pkd1^+/+^* littermate control. The Meox-2Cre recombinase activity would be expected to spare placental trophoblasts and cells derived from extra-embryonic endoderm lineages. D. In order to determine the spatial pattern of placental Meox-2 Cre activity, frozen sections of *Pkd1^cond/−^; Meox2-Cre^+^, Rosa26R* E14.5 placentas were stained with β-galactosidase (left panel) or antibodies to Pecam-1 (right panel). The dotted white line outlines the stained area. Nuclei are stained with DAPI (blue). Cre activity appears to overlap with anti-Pecam 1 staining, suggesting activity in fetal vessels that comprise the placental labyrinth layer. E. Vessel density is decreased in the labyrinth layer of *Pkd1^Meox2-Cre^* placentas compared with *Pkd1^+/+^* controls (37±2.6 branches/mm^2^ vs. 47.6±2.3 respectively) ** p<.01.

As a second complementary approach, we crossed a floxed allele of *Pkd1* (*Pkd1^cond/cond^*) to *Pkd1^+/−^, Meox2-Cre^+^* mice to produce embryos in which the floxed *Pkd1* allele was inactivated in the E6.5 embryo but not in the placental trophoblasts or extra-embryonic endoderm lineages [15], [32]. *Pkd1^cond/−^, Meox2-Cre^+^* mice also exhibited cystic kidneys, edema, and polyhydramnios (Figure 4A) but a substantial fraction survived to birth (∼16/102, expected ∼25/102). Those that survived died shortly after birth of apparent respiratory failure. The hearts of edematous *Pkd1^cond/−^, Meox2-Cre^+^* pups appeared normal with intact valves and septa, making this organ an unlikely cause of either their edema or death.

Examination of *Pkd1^cond/−^, Meox2-Cre^+^* placentas likewise showed a variable degree of improvement in placental architecture, suggesting a partial rescue. In Figure 4B and C (middle panels), the *Pkd1^cond/−^, Meox2-Cre^+^* placenta was similar to wild type, with respect to the density and complexity of placental vessels. In contrast, another placenta of the same genotype exhibited fewer fetal vessels and more dilated vascular spaces (Figure 4B and C, right panels). This was consistent with the observation that only a subset of *Pkd1^cond/−^, Meox2-Cre^+^* embryos survived to birth. On average, however, *Pkd1^cond/−^, Meox2-Cre^+^* placentas had a lower density of fetal vessels compared with controls (Figure 4E) but there was no difference in the size of any placental layer. In order to confirm the pattern of Meox2-Cre activity in the fetal placenta, we carried out crosses using mice carrying the *ROSA26R* reporter transgene [33]. The Meox2-Cre recombinase activity was restricted to the fetal portion of the placenta, overlapping with the vasculature as marked by PECAM 1 expression (Figure 4D). There was no obvious difference in Cre recombinase activity between various placentas and *Pkd1^+/cond^, Meox2-Cre^−^;Rosa26R* did not stain with β-galactosidase.

### Endothelial cell Deletion of *Pkd1* or *Pkd2* Results in Reduced Viability and Placental Defects

These findings raised the possibility that the abnormalities detected in *Pkd1* and *Pkd2* null placentas might be due to a requirement for these genes not only in trophoblasts but also in the vascular compartment of the labyrinth layer. In order to examine this possibility we ablated *Pkd1* in fetal endothelial cells using a *Tie2-Cre* transgene [34]. When we crossed *Pkd1^cond/cond^* and *Pkd1^+/−^, Tie2-Cre^+^* mice we found a reduction in live born *Pkd1^cond/−^; Tie2-Cre^+^* animals (referred to as *Pkd1^endo−^*), indicating that endothelial cell loss of polycystin-1 results in the embryonic demise of a significant fraction of animals (Table 1). If pregnancies were harvested before E18.5, however, mendelian ratios were restored.

**Table 1:** Deletion of *Pkd1* in endothelial cells.

*Pkd1^cond/cond^* vs. *Pkd1^+/−^; Tie2-Cre^+^*			
Offspring genotype	Expected ratio	Obtained Ratio(Perinatal Period)	Obtained Ratio(13.5–18.5 d.p.c)
*Pkd1cond/−; Tie2-Cre+ (Pkd1endo−)*	25%	16.9% (n = 47)	23.3% (n = 45)
*Pkd1cond/−*	25%	27.3% (n = 76)	23.8% (n = 46)
*Pkd1cond/+; Tie2-Cre+*	25%	26.2% (n = 73)	24.4% (n = 47)
*Pkd1cond/+*	25%	29.5% (n = 82)	28.5% (n = 55)

We generated a novel conditional allele of *Pkd2* in order to carry out analogous experiments (Figure S1 and S2, Table S2). Endothelial cell specific deletion of *Pkd2* (*Pkd2^endo−^*) similarly resulted in embryonic demise (Table 2). We note that in matings of *Pkd2^cond/cond^* and *Pkd2^cond/+^; Tie2-Cre^+^* mice, we consistently observed a lower than expected fraction of *Pkd2^cond/+^* progeny. Since *Pkd2^cond/+^* and *Pkd2^cond/cond^* mice are phenotypically normal and born at the expected frequencies (Table S2), the most likely explanation is that the *Pkd2* locus and the *Tie2-Cre* transgene are genetically linked but far enough apart for some recombination to occur. We detected a further reduction in live born *Pkd2^cond/cond^; Tie2-Cre^+^* (*Pkd2^endo−^*) vs. *Pkd2^cond/+^* progeny (P = .01, chi-square test) that was not present when pregnancies were harvested before E15.5. These findings suggest that there is fetal lethality of *Pkd2^endo−^* embryos superimposed on segregation distortion.

**Table 2:** Deletion of *Pkd2* in endothelial cells.

*Pkd2^cond/cond^* vs. *Pkd2^cond/+^; Tie2-Cre^+^*			
Offspring genotype	Expected ratio	Obtained Ratio(Perinatal Period)	Obtained Ratio(8.5–15.5 d.p.c)
*Pkd2cond/cond; Tie2-Cre+(Pkd2endo−)*	25%	4.3% (n = 4)	19.2% (n = 14)
*Pkd2cond/cond*	25%	35.5% (n = 33)	41.1% (n = 30)
*Pkd2cond/+; Tie2-Cre+*	25%	44.1% (n = 41)	28.7% (n = 21)
* *Pkd2cond/+*	25%	16.1% (n = 15)	11% (n = 8)

*The lower than expected percentage of *Pkd2^cond/+^* progeny suggests that *Pkd2* and the *Tie2-Cre* transgene are linked but far enough apart for some recombination to occur. There are fewer *Pkd2^cond/cond^; Tie2-Cre^+^* (*Pkd2^endo−^*) progeny compared with *Pkd2^cond/+^* (P = .01, chi-square test).

**Figure 5:**
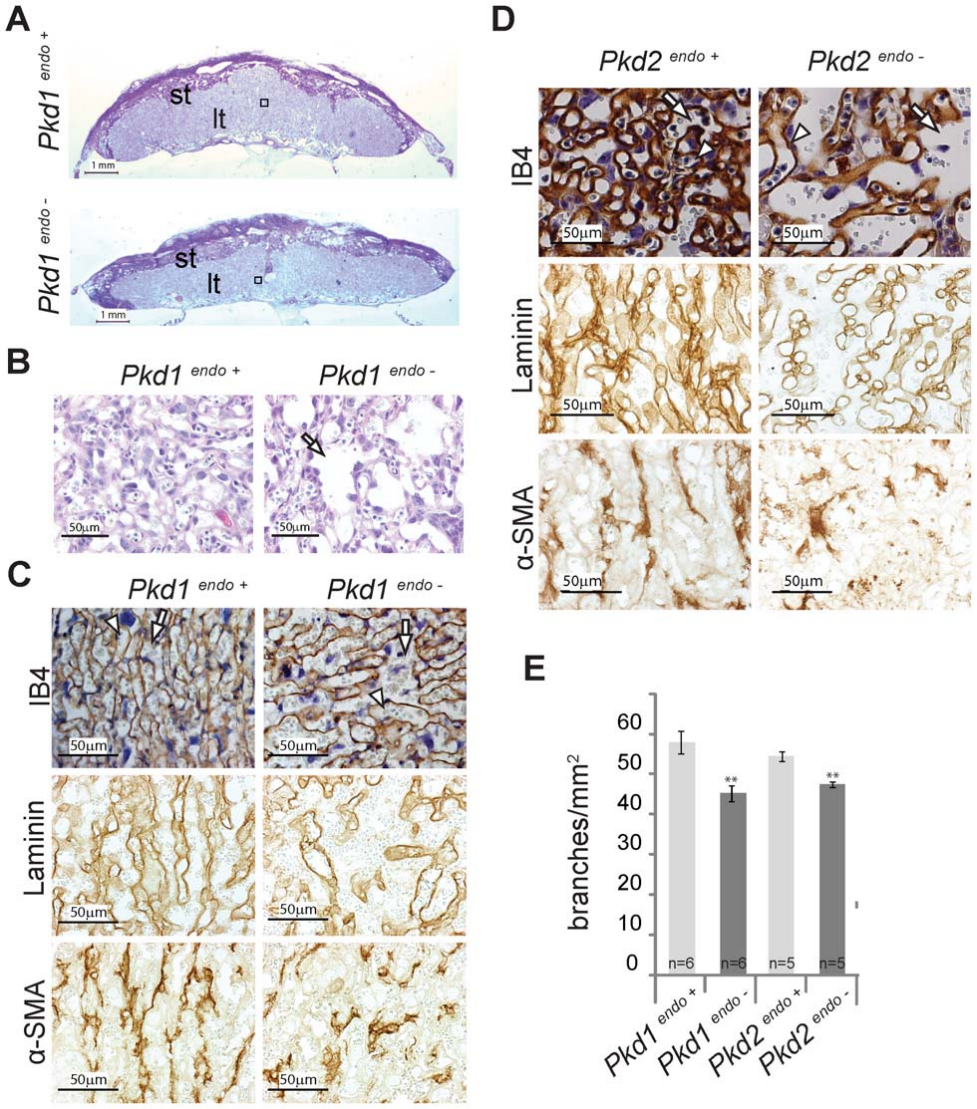
Deletion of *Pkd1* or *Pkd2* in endothelial cells results in abnormal placental histology. A, B. Representative E14.5 *Pkd2^endo^* ^***−***^ and littermate control (*Pkd2^endo^* ^***+***^) placental sections stained with PAS. Area within the square of Panel A is located in the labyrinth layer (lt), and is shown at larger magnification in Panel B. “st” = spongiotrophoblast. Arrow indicates a dilated vascular space that is likely maternal. C, D. Representative sections of E18.5 *Pkd1^endo−^* (Panel C, right), E14.5 *Pkd2^endo−^* (Panel D, right) and littermate control placentas (*Pkd1^endo+^* and *Pkd2^endo+^* left panels, C and D, respectively) stained as indicated. Both *Pkd1^endo−^* and *Pkd2^endo−^* placentas have fewer fetal vessels and investing pericytes when compared with controls. Arrowheads and arrows denote fetal vessels and maternal vascular spaces, respectively. E. Quantification of vessel density in the placental labyrinth. For *Pkd1^endo−^* vs. *Pkd1^endo+^*, the number of vessel branches is 45.3±2 and 57.9±2.9, respectively. For *Pkd2^endo−^* vs. *Pkd2^endo+^* the number of branches is 47.5±0.7 and 54.5±1.1. P **p<.01.

We examined *Pkd1^endo−^* and *Pkd2^endo−^* placentas along with littermate controls and found a phenotype similar to that described for *Pkd1* and *Pkd2* null animals (Figure 5). Isolectin B4 and laminin staining served to highlight a significant decrease in the density of fetal vessels with a simplified vascular branching pattern and dilated maternal spaces. In addition, staining for alpha-smooth muscle actin appeared more sparse and disorganized when compared with control placentas. There was no significant difference in the size of placental layers (labyrinth or spongiotrophoblast) between *Pkd1^endo−^* or *Pkd2^endo−^* placentas versus controls.

### Phenotype of mice lacking endothelial cell Polycystins

**Figure 6:**
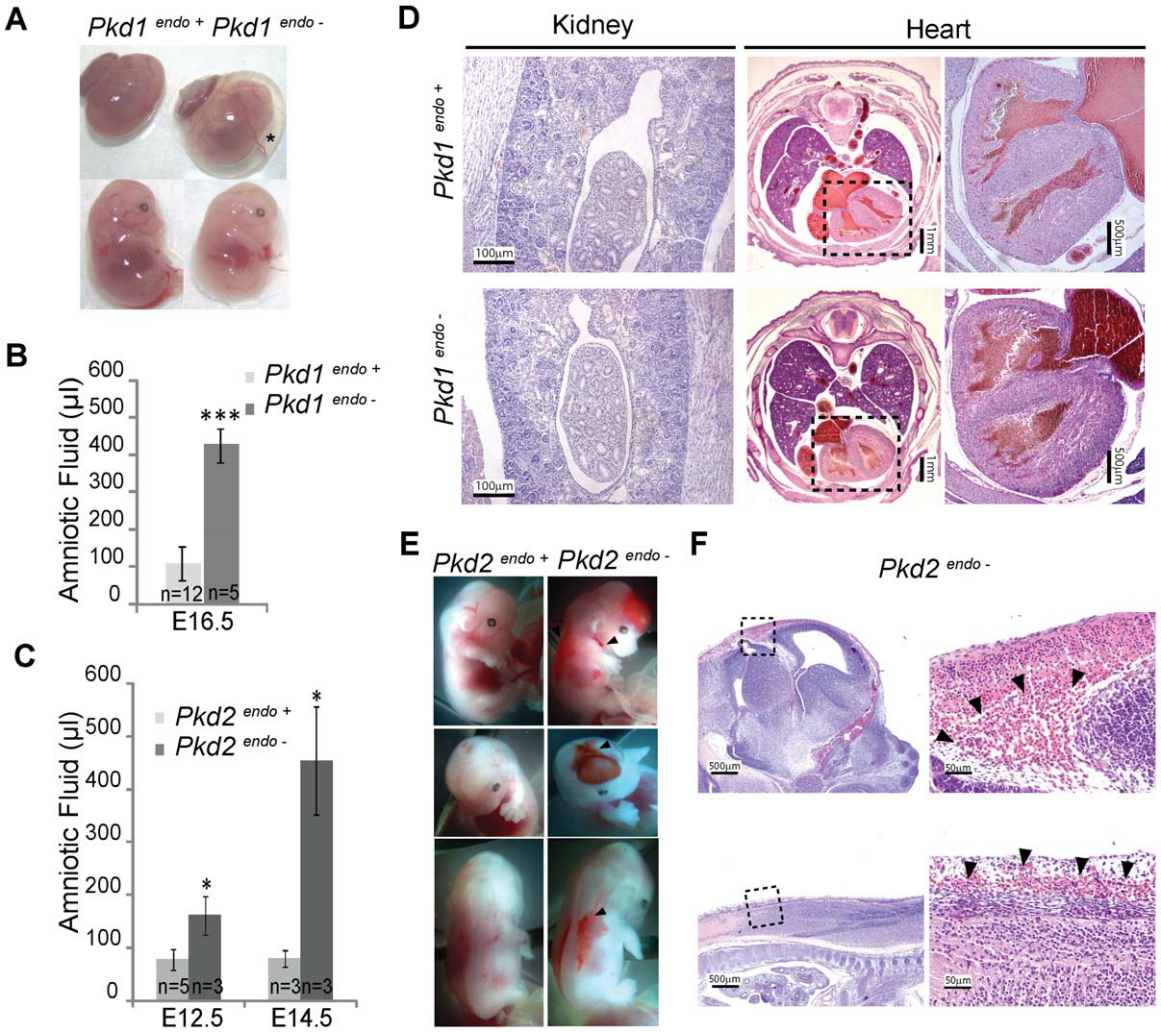
Phenotypes Associated with Endothelial cell loss of Polycystin 1 or 2. A. Gross appearance of E14.5 *Pkd1^endo−^* (right) and littermate control (left). The *Tie2-Cre^+^* (*Pkd1^endo−^*) embryo has polyhydramnios indicated by the asterisk. In the lower panel the yolk sac was removed and neither embryo has edema. B, C. Quantification of Amniotic Fluid demonstrates that both *Pkd1^endo−^* (B) and *Pkd2^endo−^* (C) embryos have significant polyhydramnios, *p<.05; *** p<.001. D. Haematoxylin-eosin staining of kidney and heart from an E16.5 *Pkd1^endo−^* embryo and control. There are no obvious abnormalities in the *Pkd1^endo−^* embryos. E. Gross appearance of an E14.5 *Pkd2^endo−^* embryo with multiple areas of hemorrhage (right). A littermate control is also shown (left). Arrowheads indicate areas of hemorrhage. F. Haematoxylin-eosin staining of hemorrhagic areas in the head (upper panel) and back (lower panel). High power view of areas in dotted square shown at right. Arrowheads indicate free red blood cells.

Unexpectedly, the phenotypic consequences of endothelial cell loss of either *Pkd1* or *Pkd2* appeared to be primarily restricted to extra-fetal tissues. *Pkd1^endo−^* and *Pkd2^endo−^* embryos universally developed polyhydramnios (Figure 6, Figure S2) and could be easily distinguished from wild type littermates. Only a small fraction of mutant animals, however, exhibited hemorrhages (Figure 6 E and F). In striking contrast to the *Pkd1* and *Pkd2* null mice, which universally had edema, none of the *Pkd1^endo−^* or *Pkd2^endo−^* embryos were edematous (Figure 5A, Figure S2F). We also failed to find any obvious cardiac or renal abnormalities in several *Pkd1^endo−^* and *Pkd2^endo−^* embryos that were sectioned (Figure 6D, Figure S2G). We maintained a cohort of the *Pkd1^endo−^* mice (n = 15) for up to one year and observed no differences in the rate of sudden death. Autopsies performed on a subset of the mutants found no obvious vascular abnormalities including aortic or intracranial aneurysms.

### Efficiency of Tie2-Cre Recombinase

**Figure 7:**
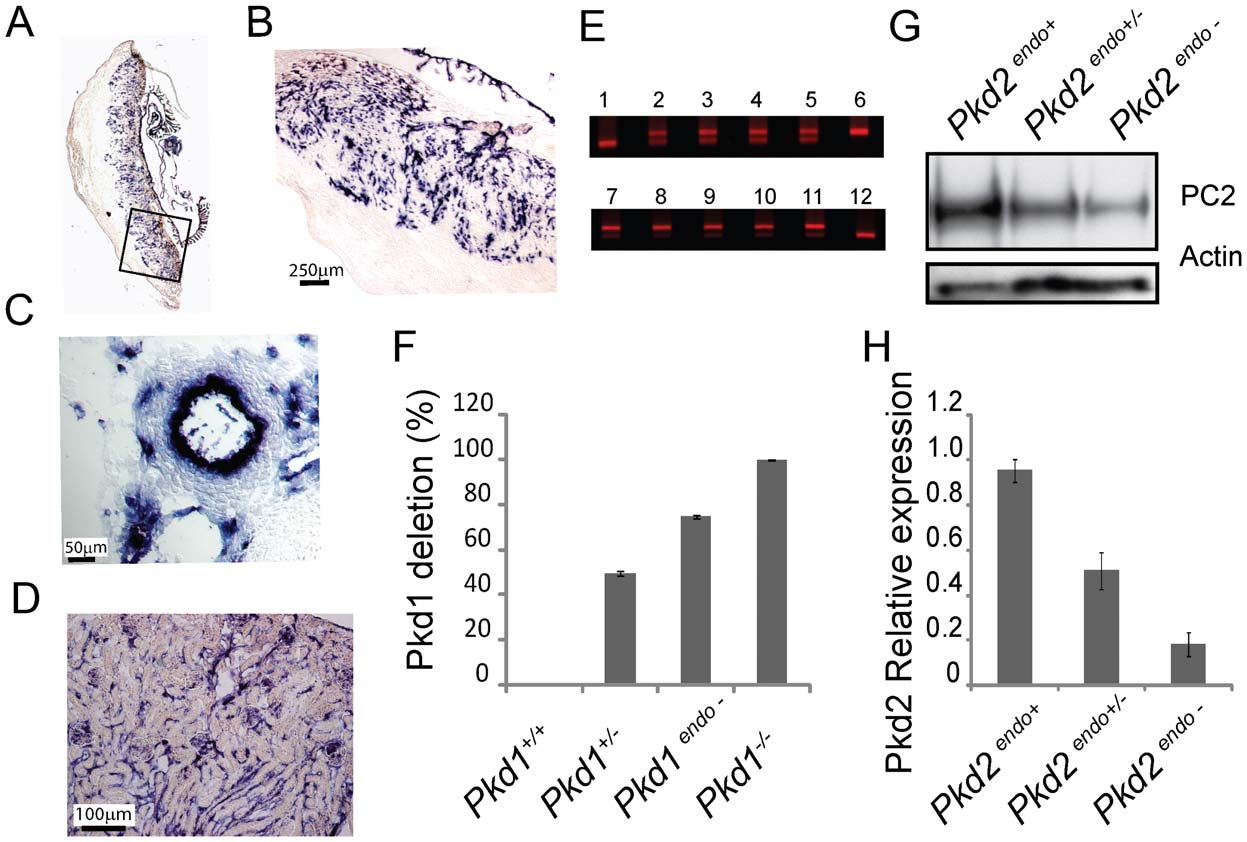
Tie2-Cre Recombinase is efficient in vivo. A–D. Alkaline Phosphatase staining of placenta (panels A, B), aorta (panel C) and kidney (panel D) from 15.5 dpc *Pkd2^cond^, Tie2-Cre^+^; Z/AP^+^*, mice. E, F. Endothelial cell DNA was prepared from N = 5 E13.5 *Pkd1^endo−^* embryos (lanes 7–11). A 3-primer PCR amplifies an 180 bp *Pkd1* wild type band (lanes 1, 12), a 250 bp deleted (null) band (lane 6) and both bands equally in *Pkd1^+/−^* DNA samples (lanes 2–5). The percent deletion of *Pkd1* was quantified in Panel F (see methods). G. Representative Western blot prepared from 13.5 dpc embryonic endothelial cells. The blot was probed with a C-terminal antibody to PC-2. Actin was used as a loading control. H. The relative expression of PC-2 was determined by correcting for loading and then normalizing to *Pkd2^endo+^*. N = 4 for *Pkd2^endo+^* and *Pkd2^endo−^*; N = 3 for *Pkd2^endo+/−^*.

Although 100% of *Pkd1^endo−^* and *Pkd2^endo−^* embryos exhibited polyhydramnios and placental abnormalities *in utero*, there was variability in the severity of the phenotype with almost 2/3 of *Pkd1^endo−^* mutants surviving to birth. One explanation for these observations could be the presence of unidentified genetic modifiers operating in the mixed background used for these studies. Alternatively phenotypic variability could be due to differences in the degree of Cre recombinase activity. In order to test for the efficiency of *Tie2-Cre* mediated recombination in endothelial cells we bred *Pkd2^cond/+^, Tie2-Cre^+^* mice with a murine line carrying the *Z/AP* transgene [35]. Tissues harvested from *Pkd2^cond^* mice carrying both *Tie2-Cre* and *Z/AP* transgenes (N = 3) demonstrated alkaline phosphatase staining in endothelial cells of all vessels examined (Figure 7A–D). Specifically the intimal layer of the aorta demonstrates intense alkaline phosphatase activity, as do most fetal vessels in the labyrinth layer of the placenta and the small vessels of the kidney and brain. As a complementary approach, we isolated endothelial cells from *Pkd1^endo−^* (n = 5) and *Pkd2^endo−^* (n = 4) embryos as well as controls and assayed for deletion using either a PCR based strategy or Western analysis (Figure 7E, F, G, H). We found that both Pkd1 and Pkd2 levels were reduced by >70%.

## Discussion

In this report we demonstrate that polycystins play a role in placental development. *Pkd1* null placentas have a reproducible defect in the labyrinth layer that is characterized by diminished complexity of fetal vascular networks along with dilated fetal and maternal spaces. We used two independent strategies to rescue *Pkd1* function in extra-embryonic placental tissue and both resulted in improved viability of *Pkd1^−/−^* embryos. We also found that targeted deletion of either *Pkd1* or *Pkd2* in endothelial cells led to a similar placental abnormality along with reduced embryonic viability. We conclude from these experiments that a defect in placentation is a likely cause of fetal demise in *Pkd1* and *Pkd2* null mice and that polycystins are required in both the trophoblast and the fetal vascular compartment of the placenta. These data are consistent with the absence of live-born humans with biallelic inactivating mutations of *PKD1* or *PKD2*.

In the mouse, the placental labyrinth layer is the main site of contact between the fetal and maternal circulation. The labyrinth consists of a highly branched fetal vascular network in close apposition to trophoblast lined maternal sinusoids [36]. As in the developing kidney, the formation of the placental labyrinth layer involves complex reciprocal interactions between trophoblasts and incipient vessels [37]. The reduced complexity of the labyrinth layer in *Pkd* mutants, suggests an additional function for the PC1/PC2 receptor-channel complex in regulating placental morphogenesis.

Interestingly, the phenotype that we observed in *Pkd* mutant placentas is similar to the defect described for PDGFB (platelet derived growth factor β) or PDGFRβ (PDGF receptor β) null placentas [29]. Placentas from these animals exhibited dilated embryonic blood vessels with fewer pericytes and trophoblasts but no reduction in size of either the labyrinth or spongiotrophoblast layers. These abnormalities are attributed to a failure of pericyte recruitment to fetal vessels, which in turn is presumed to affect the morphogenesis of complex vascular networks that comprise the placental labyrinth layer. Whether loss of either *Pkd1* or *Pkd2* in the fetal placenta affects PDGF signaling will require further investigation.

One ubiquitous feature of all *Pkd1* and *Pkd2* targeted alleles is the presence of dramatic polyhydramnios in null embryos. Surprisingly deletion of either *Pkd1* or *Pkd2* in endothelial cells alone was sufficient to recapitulate this phenotype. We were unable to identify additional renal, pulmonary or cardiac pathology that might explain these findings. Since placental membranes are thought to play a role in amniotic fluid dynamics, one possibility is that the polyhydramnios in these embryos is related to the placental abnormalities described above [38].

We note that our findings differ from those recently published using another targeted *Pkd1* null allele [39]. These authors were unable to detect a placental defect and concluded that polyhydramnios, which precedes cyst formation, is caused by abnormal proximal tubular function and excessive loss of renal solutes. Our data, however, are not consistent with this conclusion since we see polyhydramnios as early as E11.5, prior to filtration by the metanephric kidney [40]. In addition, *Pkd^endo−^* embryos lack a demonstrable renal phenotype yet they have significant polyhydramnios, making it unlikely that this is renal in origin. Finally although these authors reported that gross morphometry was no different in *Pkd1* null placentas, more sensitive measures confirm a reproducible defect in the complexity and density of fetal vessels in the placental labyrinth.

One surprising aspect of our work is the relatively limited impact of selective endothelial inactivation of either polycystin in the intact animal. Both *Pkd1* and *Pkd2* are expressed through out the cardiovascular system and null animals have a dramatic vascular phenotype manifest by edema and vascular hemorrhage [18], [20], [25]. Moreover these proteins have been detected in the primary cilia of cultured endothelial cells where they are reported to be involved in sheer stress sensing thereby activating a variety of signaling pathways that result in nitric oxide production [7], [8]. Yet, selective inactivation of either *Pkd1* or *Pkd2* in endothelial cells using a Tie-2 Cre recombinase is not sufficient to reproduce all features of vascular collapse. Most notably, *Pkd^endo−^* animals lack edema, which has been presumed to be due to leaky junctions between endothelial cells [20].

There are several conceivable explanations for these contradictory observations. It is possible, for example, that endothelial cells which escape Cre-mediated *Pkd* gene inactivation have sufficient activity to avert a more dramatic vascular phenotype. This seems unlikely given the high degree of Cre recombinase activity that we observed in all tissues examined as well as the greatly reduced level of polycystin expression that was present in endothelial cells isolated from Tie2-Cre^+^ specimens. In addition we note that others have used this Tie2-Cre recombinase line to demonstrate essential endothelial cell functions of other genes [41], [42], [43]. Alternatively, it is theoretically possible that the edematous phenotype is caused by the loss of polycystin in the lymphatic system. However, investigators have also used this Tie2-Cre recombinase to specifically delete genes required for lymphatic development [44]. We cannot exclude a primary cardiac cause for the fetal edema and hemorrhages or a circulating factor released by a cell other than one of endothelial origin. The apparent lack of significant cardiac abnormalities in severely edematous embryos, however, would make the former less likely.

One final possible explanation for our results arises from the observation that polycystins are likely involved in heterologous cell-cell interactions. The complete vascular phenotype observed in null embryos may result only when *Pkd* genes are simultaneously inactivated in multiple cell/tissue types. Alternatively, loss of polycystin in a functionally interacting cell type like smooth muscle cells may have a different effect on endothelial cell function than loss in endothelial cells themselves. Further studies using other Cre recombinases alone and in combination will be necessary to address these possibilities. While the present studies have not yet fully explained the observed vascular phenotype, they do reveal the unexpectedly complex role these proteins likely play in maintaining vascular integrity.

## Materials and Methods

### Ethics Statement

All studies were performed using protocols approved by the University Animal Care and Use Committee (Protocol MO05M318), and mice were housed and cared in pathogen-free facilities accredited by the American Association for the Accreditation of Laboratory Animal Care and meet federal (NIH) guidelines for the humane and appropriate care of laboratory animal.

### Endothelial Cell Ablation of Pkd1 or Pkd2

*Pkd1* null alleles and conditional alleles have been previously described [15], [45]. We inactivated *Pkd1* or *Pkd2* in endothelial cells using transgenic mice expressing Cre recombinase under the control of the *Tie2* promoter (*Tie2-Cre* line; Masashi Yanagisawa, UT Southwestern) [34]. *Pkd1^+/−^* heterozygotes were crossed to *Tie2-Cre* transgenic mice to generate *Pkd1^+/−^, Tie2-Cre^+^* males. These males were crossed to *Pkd1^cond/cond^* females to generate *Pkd1^cond/−^, Tie2-Cre^+^* mice (*Pkd1^endo−^*) mice. For *Pkd2*, we mated *Pkd2^cond/cond^* homozygous females to the *Pkd2^cond/+^, Tie2-Cre^+^* males to create *Pkd2^cond/cond^, Tie2-Cre^+^* mice (*Pkd2^endo−^*). Genotyping was performed from tail DNA using the REDExtract-N-Amp tissue PCR Kit (SIGMA) according to the manufacturer's protocol. *Pkd1^cond^* and null alleles were genotyped as previously described [15]. *Pkd2* alleles were genotyped as described in supplementary methods S1.

### Quantitative PCR Analysis

Total RNA was isolated from individual placentas using the Qiagen RNA extraction kit. Five micrograms of total RNA was used for first strand cDNA synthesis using Superscript II (Invitrogen). Quantitative PCR (*Stratagene Mx3005SP*) for *Pkd1* and *Pkd2* expression was performed using the SYBR green PCR master mix (Applied Biosystems). Samples were run in triplicate and an RT negative sample was used to control for DNA contamination. A water control was also included. Annealing temperatures and primer sequences are as follows: *Pkd1*, 60°C: 5′- TCTGGATGGGCTTCAGCAA and 5′- AGCGGGAAGGCAGTGGAT *;* *Pkd2*, *60°C: 5′- AGACTTCTCGGTGTATAACGCAAA and 5′- CACCCGTTGCTGGGAACT. Values were normalized to those of RPL13 (ribosomal protein L13).*

### Immunoprecipitation/Western Blotting

Samples were homogenized in lysis buffer (20 mM Na phosphate (pH 7.2), 150 mM NaCl, 1 mm EDTA, 10% glycerol, 1% Triton X-100 and protease inhibitor cocktail (Roche). The homogenate was incubated for 1 hour on ice and cleared of debris by centrifugation at 17, 000× g for 10 min at 4°C. Six milligrams of protein in 1 ml was used for immunoprecipitation (IP) with 1 ul of a polyclonal chicken antibody (chicken anti-CC) directed at the mouse PC1 C-terminus. Western blots were probed with rabbit antisera directed against the PC2 C-terminus (PC2 anti-CT, 1∶1000) or the PC1 C-terminus (PC1 anti-CC, 1∶1000). Antisera to PC1 and PC2 have been described [46], [47].

### Reporter Analysis

Cre mediated recombination was visualized with either the *ROSA26R* reporter line, which expresses lacZ upon Cre mediated activation or the Z/AP double reporter line [33], [35]. Frozen sections were prepared for LacZ and alkaline phosphatase staining using the method of Lobe et al. [35].

### Immunohistochemistry

Placental sections were prepared and stained with peroxidase conjugated IB4 (Sigma L5391) using published methods [29]. Placental pericytes were stained with the α-smooth muscle actin immunohistology kit (IMMH2, from Sigma) using the manufacturer's protocol. Anti-Laminin antibodies (Sigma L9393) were used at a final concentration of 1∶400. Frozen placental sections were stained with Anti-Pecam antibody (BD Pharmingen, MEC13.3) diluted to 1∶500. Indirect fluorescence microscopy was performed using a Nikon Eclipse E600 and images were captured using a SPOT-RT monochromic camera (SPOT Diagnostic Instruments). At least 4 sections were examined for each genotype except in the case of tetraploid rescue.

### Placental Morphometry

At least 4 independent pairs of placentas were used for comparison: 1) *Pkd1^−/−^* vs. *Pkd1^+/+^* at E12.5 2) *Pkd1^endo−^* vs. *Pkd1^endo+^* at E18.5 3) *Pkd2^endo−^* vs. *Pkd2^endo+^* at E14.5 and 4) *Pkd1^cond/−^, Meox2-Cre^+^* vs. *Pkd1^cond/−^, Meox2-Cre^−^* at E14.5. Placentas were fixed, paraffin embedded, hemisected and 5 µm sections were prepared. Two separate placental sections representing the middle section of the placenta were hybridized with a *Tpbp* riboprobe (Dr. JC Cross, University of Calgary) to distinguish the spongiotrophoblast and the overlying the labyrinth region. Digital photographs at 1X were taken and NIH Image J software was used to measure total placental area, labyrinth and spongiotrophoblast areas. Three independent measurements were taken for each layer and section. In order to assay vessel density, hemisected placentas were stained with IB4 as above. Six to twelve photographs at 20X magnification were taken from two different sections (middle region) representing more than 90% of the labyrinth. The vessels were counted using NIH Image J software that equally distributes 208 crosses (comprising a .96 mm^2^ grid) over the photograph. A blinded observer counted the vessels touching each cross. Comparisons between groups were performed using the two-tailed unpaired Student's t-test (Excel, Microsoft).

### Endothelial Cell Isolation and Analysis of Cre Deletion

Minced E13.5 embryos were digested with collagenase II (2 mg/ml) and DNase I to generate a single cell suspension. After centrifuging, the cell pellet was washed, and passed through a 40 um cell strainer. The cells suspension was incubated with Anti-Pecam (CD31, BD Biosciences) coated Dynabeads (Dynal) per manufacturer's recommendations. The beads were separated using a magnetic particle concentrator. Western analysis for PC2 was performed and the relative expression was calculated by correcting for loading and then normalizing to *Pkd2^endo+^* (100% expression). For *Pkd1*, genomic DNA was prepared from endothelial cells and a 3 primer PCR amplification for 25 cycles was performed with SYBR green Master Mix (Applied Biosystems) using primers and conditions previously described [15]. Both *Pkd1* wild type (WT) and conditional alleles give a180 bp band while the null or deleted (KO) allele yields a 250 bp band. The relative intensity of the two bands was determined using the Molecular Imager System® Pharos FxPlus™ (Bio-Rad) and used to calculate the relative *Pkd1* deletion (intensity KO band÷intensity WT band + KO band).

## Supporting Information

Supplementary Methods S1 (0.05 MB DOC)

Figure S1 *Generation of Floxed and Mutant alleles of Pkd2*. A. Schematic representation of the *Pkd2* targeting construct (TC), *Pkd2* locus (Gene) and resulting allelic series. *Pkd2^flox11-13^* (*Pkd2^tm1Tjw1^*) refers to the floxed allele. *Pkd2^Δneo^* (*Pkd2^tm1.1Tjw^*) results from deletion of the Neomycin cassette. We refer to this allele as *Pkd2^cond^* in the body of the paper. Deletion with Cre recombinase yields the mutant allele, *Pkd2^Δ11-13^* (*Pkd2^tm1.2Tjw^*). This is referred to as *Pkd2^−^* in the body of the paper. The restriction maps for *AflII* and *BglII* are as indicated. The location of PCR primers a, b, and c in the various *Pkd2* alleles is shown along with the size of the corresponding PCR products. The red and yellow triangles represent *loxP* and *Frt* sites, respectively and grey ovals correspond to exons. B. Genomic Southern showing germ line transmission of *Pkd2^flox11-13^* allele. The “+” signifies a mouse carrying the targeted allele and “-” is a wild type littermate. DNA from the offspring of highly chimeric mice was digested with either *AflII* (top) or *BglII* (bottom) and hybridized with 5′ or 3′ probes (position depicted in panel A). In each case the probe detects the wild type band and a larger band as expected for the appropriately targeted locus. Internal probes (position depicted in panel A) only hybridize to the bands recognized by the external probes indicating that there were no random integration events. C. Genotyping with a 3-primer PCR strategy. Primers a, b and c were used to identify wild type, floxed and deleted alleles. A representative ethidium bromide stained gel is shown. Primers a and b amplify a 232 bp band from the wild type allele and a 318 bp band in the floxed allele. Primers a and c are far apart and do not amplify a product from genomic DNA in either allele. In the deleted allele the primer b site is lost and a 143 bp band is amplified from primers a and c. D. The *Pkd2^Δ11-13^* mutant allele is transcribed. The intron/exon structure of wild type and mutant (*Pkd2^Δ11-13^*) alleles along with the location of primers f, h, i are shown schematically. Cre recombinase is predicted to result in a frame shift and stop codon “UAG” in exon 14. RTPCR products amplified from embryos of the indicated genotypes are shown. “-” is the water control. Primers f and i yield a 900 bp and a 496 bp product from wild type and and mutant alleles respectively. Both bands are seen in a heterozygote, *Pkd2^Neo3/Δ11-13^*. The faint band indicated by the asterisk is likely due to heteroduplex formation. Primers h and i, contained within exon 15, amplify a 105 bp band from both alleles. E. The *Pkd2^Δ11-13^* mutant allele does not yield protein. Total lysates derived from embryos of the indicated genotypes were used to prepare Western blots. Both the N-terminal and C-terminal PC-2 antibodies fail to detect polycystsin-2 in the *Pkd2^Δ11-13^* mutant. Alpha tubulin was used as a loading control. F. Cell lysates were prepared from various embryos and human PKD2 transfected HEK cells (positive control). PC-2 was immunoprecipitated and then detected with either N-terminal or C-terminal antibodies as indicated. PC-2 cannot be immunoprecipitated from *Pkd2^Δ11-13^* homozygotes with either the N-terminal or C-terminal antibody. The star indicates a non-specific band that is seen when the N-terminal antibody is used for both IP and Western blot. This non-specific band was not visualized when total lysates were probed with the N-terminal antibody (data not shown).
(3.61 MB TIF)

Figure S2 *Phenotypes of Floxed and Mutant alleles of Pkd2*. A. *Pkd2^Δ11-13/+^* heterozygotes were bred and pregnancies harvested at various time points as indicated. The gross phenotypes of *Pkd2^Δ11-13/Δ11-13^* embryos are demonstrated. The asterisk, “*”, indicates edema, Arrowheads denote areas of hemorrhage. The E16.5 embryos were dead and in the process of being resorbed. B. Hematoxylin and eosin staining of kidney and pancreas sections of normal (top) and mutant (bottom) E15.5 embryos. C. *Pkd2^cond/Δ11-13^; Meox2-Cre^+^* mice were generated using standard breeding protcols. The top left panel shows the general appearance of a P0 *Pkd2^cond/Δ11-13^; Meox2-Cre^+^* neonate with edema (asterisk). On the right, necropsy shows a cystic kidney. In the bottom panels, histopathology confirms the presence of cysts in the kidney (K) and pancreas (P). “Cy” indicates cysts. D. The top left panel shows the general appearance of another P0 *Pkd2^cond/Δ11-13^; Meox2-Cre^+^* neonate. Necropsy of the pup, which died shortly after birth, demonstrates situs inversus (top middle and right panels). The areas in the red squares are magnified in the lower panels. Right-sided stomach and spleen (left lower panel) as well as dextrocardia (right lower panel, arrow) are seen. L: liver, P: pancreas, K: kidney, S: stomach, Sp: spleen, Cy: cyst. E. Cysts derive from all tubular segments. Cystic kidneys from the embryo in panel A stained with markers for proximal tubule (LTL, green), thick ascending limb (Tamm Horsfall, red) collecting duct (aquaporin-2, red) and Nuclei (DAPI, blue). F. Gross appearance of an E14.5 *Pkd2^endo−^* embryo and littermate control. The *Pkd2^endo−^* embryo has polyhydramnios indicated by the asterisk. At the right, the yolk sac was removed and neither embryo has edema. G. Haematoxylin-eosin staining of kidney and heart from an E13.5 *Pkd2^endo−^* embryo and littermate control. As in *Pkd1^endo−^*, embryos, there were no obvious histopathology abnormalities in the animals bearing the Tie-2 Cre recombinase (*Pkd2^endo−^*).
(9.42 MB TIF)

Table S1 *Genotypes resulting from Pkd1^+/−^ Intercrosses*.
(0.11 MB PDF)

Table S2 *Genotypes resulting from mating of Pkd2 allele*.
(0.15 MB PDF)

## References

[1] Gabow PA (1993). Autosomal dominant polycystic kidney disease.. N Engl J Med.

[2] Parfrey PS, Bear JC, Morgan J, Cramer BC, McManamon PJ (1990). The diagnosis and prognosis of autosomal dominant polycystic kidney disease.. N Engl J Med.

[3] Pirson Y Extrarenal manifestations of autosomal dominant polycystic kidney disease.. Adv Chronic Kidney Dis.

[4] Harris PC, Torres VE (2009). Polycystic kidney disease.. Annu Rev Med.

[5] Gallagher AR, Germino GG, Somlo S Molecular advances in autosomal dominant polycystic kidney disease.. Adv Chronic Kidney Dis.

[6] Nauli SM, Alenghat FJ, Luo Y, Williams E, Vassilev P (2003). Polycystins 1 and 2 mediate mechanosensation in the primary cilium of kidney cells.. Nat Genet.

[7] Nauli SM, Kawanabe Y, Kaminski JJ, Pearce WJ, Ingber DE (2008). Endothelial cilia are fluid shear sensors that regulate calcium signaling and nitric oxide production through polycystin-1.. Circulation.

[8] AbouAlaiwi WA, Takahashi M, Mell BR, Jones TJ, Ratnam S (2009). Ciliary polycystin-2 is a mechanosensitive calcium channel involved in nitric oxide signaling cascades.. Circ Res.

[9] Baert L (1978). Hereditary polycystic kidney disease (adult form): a microdissection study of two cases at an early stage of the disease.. Kidney Int.

[10] Watnick T, He N, Wang K, Liang Y, Parfrey P (2000). Mutations of PKD1 in ADPKD2 cysts suggest a pathogenic effect of trans-heterozygous mutations.. Nat Genet.

[11] Pei Y, Watnick T, He N, Wang K, Liang Y (1999). Somatic PKD2 mutations in individual kidney and liver cysts support a “two-hit” model of cystogenesis in type 2 autosomal dominant polycystic kidney disease.. J Am Soc Nephrol.

[12] Peters DJ, Sandkuijl LA (1992). Genetic heterogeneity of polycystic kidney disease in Europe.. Contrib Nephrol.

[13] Qian F, Watnick TJ, Onuchic LF, Germino GG (1996). The molecular basis of focal cyst formation in human autosomal dominant polycystic kidney disease type I.. Cell.

[14] Wu G, D'Agati V, Cai Y, Markowitz G, Park JH (1998). Somatic inactivation of Pkd2 results in polycystic kidney disease.. Cell.

[15] Piontek KB, Huso DL, Grinberg A, Liu L, Bedja D (2004). A functional floxed allele of Pkd1 that can be conditionally inactivated in vivo.. J Am Soc Nephrol.

[16] Lu W, Fan X, Basora N, Babakhanlou H, Law T (1999). Late onset of renal and hepatic cysts in Pkd1-targeted heterozygotes.. Nat Genet.

[17] Piontek K, Menezes LF, Garcia-Gonzalez MA, Huso DL, Germino GG (2007). A critical developmental switch defines the kinetics of kidney cyst formation after loss of Pkd1.. Nat Med.

[18] Boulter C, Mulroy S, Webb S, Fleming S, Brindle K (2001). Cardiovascular, skeletal, and renal defects in mice with a targeted disruption of the Pkd1 gene.. Proc Natl Acad Sci U S A.

[19] Lu W, Peissel B, Babakhanlou H, Pavlova A, Geng L (1997). Perinatal lethality with kidney and pancreas defects in mice with a targetted Pkd1 mutation.. Nat Genet.

[20] Kim K, Drummond I, Ibraghimov-Beskrovnaya O, Klinger K, Arnaout MA (2000). Polycystin 1 is required for the structural integrity of blood vessels.. Proc Natl Acad Sci U S A.

[21] Muto S, Aiba A, Saito Y, Nakao K, Nakamura K (2002). Pioglitazone improves the phenotype and molecular defects of a targeted Pkd1 mutant.. Hum Mol Genet.

[22] Wu G, Markowitz GS, Li L, D'Agati VD, Factor SM (2000). Cardiac defects and renal failure in mice with targeted mutations in Pkd2.. Nat Genet.

[23] Pennekamp P, Karcher C, Fischer A, Schweickert A, Skryabin B (2002). The ion channel polycystin-2 is required for left-right axis determination in mice.. Curr Biol.

[24] Paterson AD, Wang KR, Lupea D, St George-Hyslop P, Pei Y (2002). Recurrent fetal loss associated with bilineal inheritance of type 1 autosomal dominant polycystic kidney disease.. Am J Kidney Dis.

[25] Guay-Woodford LM (2003). Murine models of polycystic kidney disease: molecular and therapeutic insights.. Am J Physiol Renal Physiol.

[26] Qian Q, Li M, Cai Y, Ward CJ, Somlo S (2003). Analysis of the polycystins in aortic vascular smooth muscle cells.. J Am Soc Nephrol.

[27] Gonzalez-Perrett S, Kim K, Ibarra C, Damiano AE, Zotta E (2001). Polycystin-2, the protein mutated in autosomal dominant polycystic kidney disease (ADPKD), is a Ca2+-permeable nonselective cation channel.. Proc Natl Acad Sci U S A.

[28] Ong AC, Ward CJ, Butler RJ, Biddolph S, Bowker C (1999). Coordinate expression of the autosomal dominant polycystic kidney disease proteins, polycystin-2 and polycystin-1, in normal and cystic tissue.. Am J Pathol.

[29] Ohlsson R, Falck P, Hellstrom M, Lindahl P, Bostrom H (1999). PDGFB regulates the development of the labyrinthine layer of the mouse fetal placenta.. Dev Biol.

[30] Allen E, Piontek KB, Garrett-Mayer E, Garcia-Gonzalez M, Gorelick KL (2006). Loss of polycystin-1 or polycystin-2 results in dysregulated apolipoprotein expression in murine tissues via alterations in nuclear hormone receptors.. Hum Mol Genet.

[31] James RM, Klerkx AH, Keighren M, Flockhart JH, West JD (1995). Restricted distribution of tetraploid cells in mouse tetraploid< =  = >diploid chimaeras.. Dev Biol.

[32] Tallquist MD, Soriano P (2000). Epiblast-restricted Cre expression in MORE mice: a tool to distinguish embryonic vs. extra-embryonic gene function.. Genesis.

[33] Soriano P (1999). Generalized lacZ expression with the ROSA26 Cre reporter strain.. Nat Genet.

[34] Kisanuki YY, Hammer RE, Miyazaki J, Williams SC, Richardson JA (2001). Tie2-Cre transgenic mice: a new model for endothelial cell-lineage analysis in vivo.. Dev Biol.

[35] Lobe CG, Koop KE, Kreppner W, Lomeli H, Gertsenstein M (1999). Z/AP, a double reporter for cre-mediated recombination.. Dev Biol.

[36] Watson ED, Cross JC (2005). Development of structures and transport functions in the mouse placenta.. Physiology (Bethesda).

[37] Rossant J, Cross JC (2001). Placental development: lessons from mouse mutants.. Nat Rev Genet.

[38] Beall MH, van den Wijngaard JP, van Gemert MJ, Ross MG (2007). Regulation of amniotic fluid volume.. Placenta.

[39] Ahrabi AK, Jouret F, Marbaix E, Delporte C, Horie S Glomerular and proximal tubule cysts as early manifestations of Pkd1 deletion.. Nephrol Dial Transplant.

[40] Zamboni L, Upadhyay S (1981). Ephemeral, rudimentary glomerular structures in the mesonephros of the mouse.. Anat Rec.

[41] Cattelino A, Liebner S, Gallini R, Zanetti A, Balconi G (2003). The conditional inactivation of the beta-catenin gene in endothelial cells causes a defective vascular pattern and increased vascular fragility.. J Cell Biol.

[42] Allende ML, Yamashita T, Proia RL (2003). G-protein-coupled receptor S1P1 acts within endothelial cells to regulate vascular maturation.. Blood.

[43] Fu J, Gerhardt H, McDaniel JM, Xia B, Liu X (2008). Endothelial cell O-glycan deficiency causes blood/lymphatic misconnections and consequent fatty liver disease in mice.. J Clin Invest.

[44] Srinivasan RS, Dillard ME, Lagutin OV, Lin FJ, Tsai S (2007). Lineage tracing demonstrates the venous origin of the mammalian lymphatic vasculature.. Genes Dev.

[45] Bhunia AK, Piontek K, Boletta A, Liu L, Qian F (2002). PKD1 induces p21(waf1) and regulation of the cell cycle via direct activation of the JAK-STAT signaling pathway in a process requiring PKD2.. Cell.

[46] Yu S, Hackmann K, Gao J, He X, Piontek K (2007). Essential role of cleavage of Polycystin-1 at G protein-coupled receptor proteolytic site for kidney tubular structure.. Proc Natl Acad Sci U S A.

[47] Boletta A, Qian F, Onuchic LF, Bhunia AK, Phakdeekitcharoen B (2000). Polycystin-1, the gene product of PKD1, induces resistance to apoptosis and spontaneous tubulogenesis in MDCK cells.. Mol Cell.

