# Supplementary material for: Pkd1 and Pkd2 Are Required for Normal Placental Development
Source: PLoS One. 2010 Sep 16;5(9):e12821. doi: 10.1371/journal.pone.0012821 (PMC2940908; doi:10.1371/journal.pone.0012821)
Supplement: Supplementary Methods S1 — (0.05 MB DOC) [file pone.0012821.s001.doc]

**Supplementary Information.**

**Supplementary Material and Methods.**

*Generation of Pkd2^flox11-13^ mice:* The targeting construct, generated using genomic PCR and standard cloning strategies, was designed to produce deletion of *Pkd2* exons 11-13 (Fig. S1). A single *lox P* site was inserted into intron 10. In addition, a Pgk-Neo selection cassette flanked by *FRT* sites and another *lox P* site (gift of Dr. Gail Martin) was introduced into intron 13(1). The construct was sequenced prior to injection in order to verify orientation of the lox P sites and coding sequence. Primers used to generate the targeting construct are available on request.

The linearized targeting construct was electroporated into 129SV/J cells (Ingenious Targeting Laboratory, Stonybrook, NY). Approximately 200 ES clones were screened and one clone (of three), verified to have a single, legitimate targeting event, with both *lox P* sites intact, was injected into C57BL/6 blastocysts (Ingenious Targeting Laboratory). Highly chimeric male mice were obtained and crossed to 129SvEv females to obtain the *Pkd2 ^Tm1Tjw^* (*Pkd2^flox11-13^*) line. Mice that were homozygous for the *Pkd2^flox11-13^* allele had significantly reduced viability in het-het matings (Table S2). The neomycin gene was deleted by breeding *Pkd2^flox11-13^* mice to a mouse line expressing the *Flp1* recombinase gene under the direction of the human *ACTB* promoter (B6;J- strain TgN[ACTFLPe]9205Dym, gift of Dr. Dynecki) to generate *Pkd2^tm1.1Tjw^* (*Pkd2^flox11-13∆neo^* also referred to *Pkd2^cond^* in the body of the text). After deletion of the neomycin gene, *Pkd2^flox11-13∆neo^* homozygotes were viable and fertile. We generated mice with germ line excision of exons 11-13, *Pkd2^∆11-13^* (*Pkd2^tm1.2Tjw^*), by crossing *Pkd2^flox11-13∆neo^* mice to a deleter strain (*Meox-Cre B6.129S4-Meox2 ^tm1[cre]Sor^*), which induces Cre-mediated recombination at a high rate in somatic tissues (Jackson Laboratories).

*Genotyping:*  *Pkd2^flox11-13∆neo^*, wild type and *Pkd2^∆11-13^* alleles were distinguished using a 3 primer PCR strategy: Primer a: 5’-CCTTTCCTCTGTGTTCTGGGGAG, Primer b: GTTTGATGCTTAGCAGATGGC and Primer c: 5’-CTGACAGGCACCTACAGAACAGTG (Figure 1A). PCR products were resolved on 2% 3:1 NuSieve agarose gels, yielding products of 318, 232 and 143 base pairs, respectively (Figure 1C). The Cre transgene was detected using the following primers: Forward: 5’-ATTGCTGTCACTTGGTCGTGGC and Reverse: 5’-GGAAAATGCTTCTGTCCGTTTGC. Presence of the Z/AP transgene was determined using Forward Primer 5’-ATGTTCGACGACGCCATTG and Reverse primer 5’-CACTGCTGACTGCTGCCGATA. Specific PCR conditions are available upon request.

*Reverse Transcription PCR:* Total RNA was isolated using the Qiagen RNA extraction kit. 5μg of total RNA was used for first strand cDNA synthesis using Superscript II (Invitrogen). *Pkd2* transcripts in Supplementary Figure 5 were amplified using forward primers in exon 10 (10F:5’-CAGCAGAAAGCAGAAATGG) or exon 15 (5’-GGAAGTGGAAATGGAAGTGCTAAC) in combination with a reverse primer in exon 15 (15R:5’CTTATCATTGTCGTACTGGACAGCC).

*Immunoprecipitation/Western Blotting:* Total protein was isolated from embryos, placentas or HEK cells transfected with a wild-type *Pkd2* construct (2). Tissue was homogenized in lysis buffer (20mM Na phosphate (pH 7.2), 150mM NaCl, 1mm EDTA, 10% glycerol, 1% Triton X-100 and protease inhibitor cocktail (Roche). For immunoprecipitation (IP), the cleared lysate was incubated with (0.5ul/ml) of antisera at 4°C and then with Protein G Sepharose beads. The IPs for polycystin-2 (PC2) were performed with either an antibody that recognizes an epitope near its carboxyl (Santa Cruz, SC2833) or amino termini (Zymed). The IP products and lysates were resolved on a 3-8% NuPage gel (Invitrogen) and then electroblotted to a polyvinylidene difluoride membrane. The blots were probed with antisera to: the PC2 C-terminus (PC2 CT, 1:1000), to the N-terminus (1:250, Zymed), to α-tubulin (Sigma) or actin (Santa Cruz). PC2 CT antibody has been previously characterized (3)**.**

**Supplementary References:**

1. Meyers EN, Lewandoski M, & Martin GR (1998) An Fgf8 mutant allelic series generated by Cre- and Flp-mediated recombination. *Nat Genet* 18(2):136-141.

2. Hanaoka K*, et al.* (2000) Co-assembly of polycystin-1 and -2 produces unique cation-permeable currents. *Nature* 408(6815):990-994.

3. Boletta A*, et al.* (2000) Polycystin-1, the gene product of PKD1, induces resistance to apoptosis and spontaneous tubulogenesis in MDCK cells. *Mol Cell* 6(5):1267-1273.
