# Supplementary figures and images for: Pkd1 and Pkd2 Are Required for Normal Placental Development
Source: PLoS One. 2010 Sep 16;5(9):e12821. doi: 10.1371/journal.pone.0012821 (PMC2940908; doi:10.1371/journal.pone.0012821)

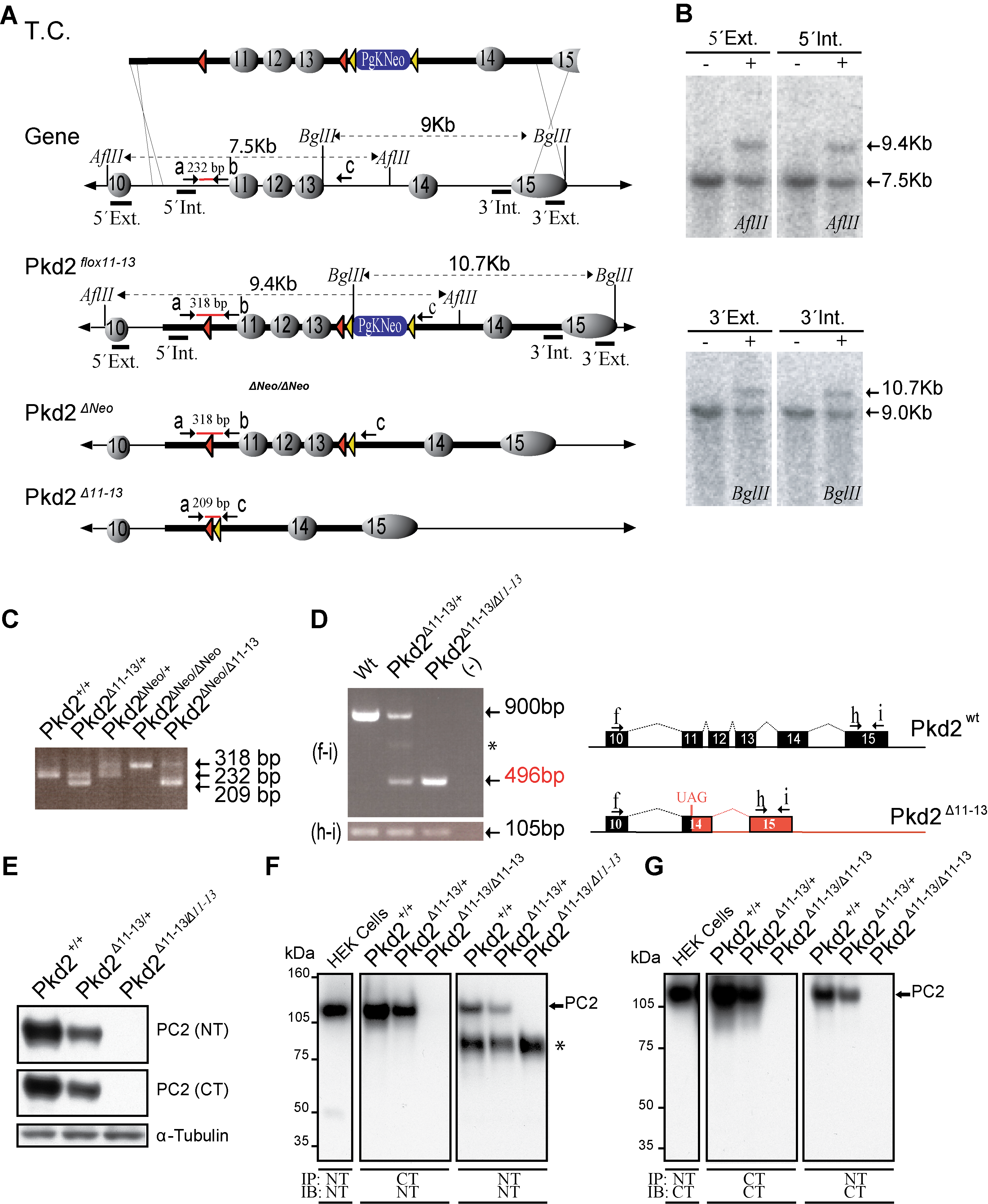

Supplement: Figure S1 — Generation of Floxed and Mutant alleles of Pkd2. A. Schematic representation of the Pkd2 targeting construct (TC), Pkd2 locus (Gene) and resulting allelic series. Pkd2flox11-13 (Pkd2tm1Tjw1) refers to the floxed allele. Pkd2Δneo (Pkd2tm1.1Tjw) results from deletion of the Neomycin cassette. We refer to this allele as Pkd2cond in the body of the paper. Deletion with Cre recombinase yields the mutant allele, Pkd2Δ11-13 (Pkd2tm1.2Tjw). This is referred to as Pkd2− in the body of the paper. The restriction maps for AflII and BglII are as indicated. The location of PCR primers a, b, and c in the various Pkd2 alleles is shown along with the size of the corresponding PCR products. The red and yellow triangles represent loxP and Frt sites, respectively and grey ovals correspond to exons. B. Genomic Southern showing germ line transmission of Pkd2flox11-13 allele. The “+” signifies a mouse carrying the targeted allele and “-” is a wild type littermate. DNA from the offspring of highly chimeric mice was digested with either AflII (top) or BglII (bottom) and hybridized with 5′ or 3′ probes (position depicted in panel A). In each case the probe detects the wild type band and a larger band as expected for the appropriately targeted locus. Internal probes (position depicted in panel A) only hybridize to the bands recognized by the external probes indicating that there were no random integration events. C. Genotyping with a 3-primer PCR strategy. Primers a, b and c were used to identify wild type, floxed and deleted alleles. A representative ethidium bromide stained gel is shown. Primers a and b amplify a 232 bp band from the wild type allele and a 318 bp band in the floxed allele. Primers a and c are far apart and do not amplify a product from genomic DNA in either allele. In the deleted allele the primer b site is lost and a 143 bp band is amplified from primers a and c. D. The Pkd2Δ11-13 mutant allele is transcribed. The intron/exon structure of wild type and mutant (Pkd2Δ11-13) [file pone.0012821.s002.tif]

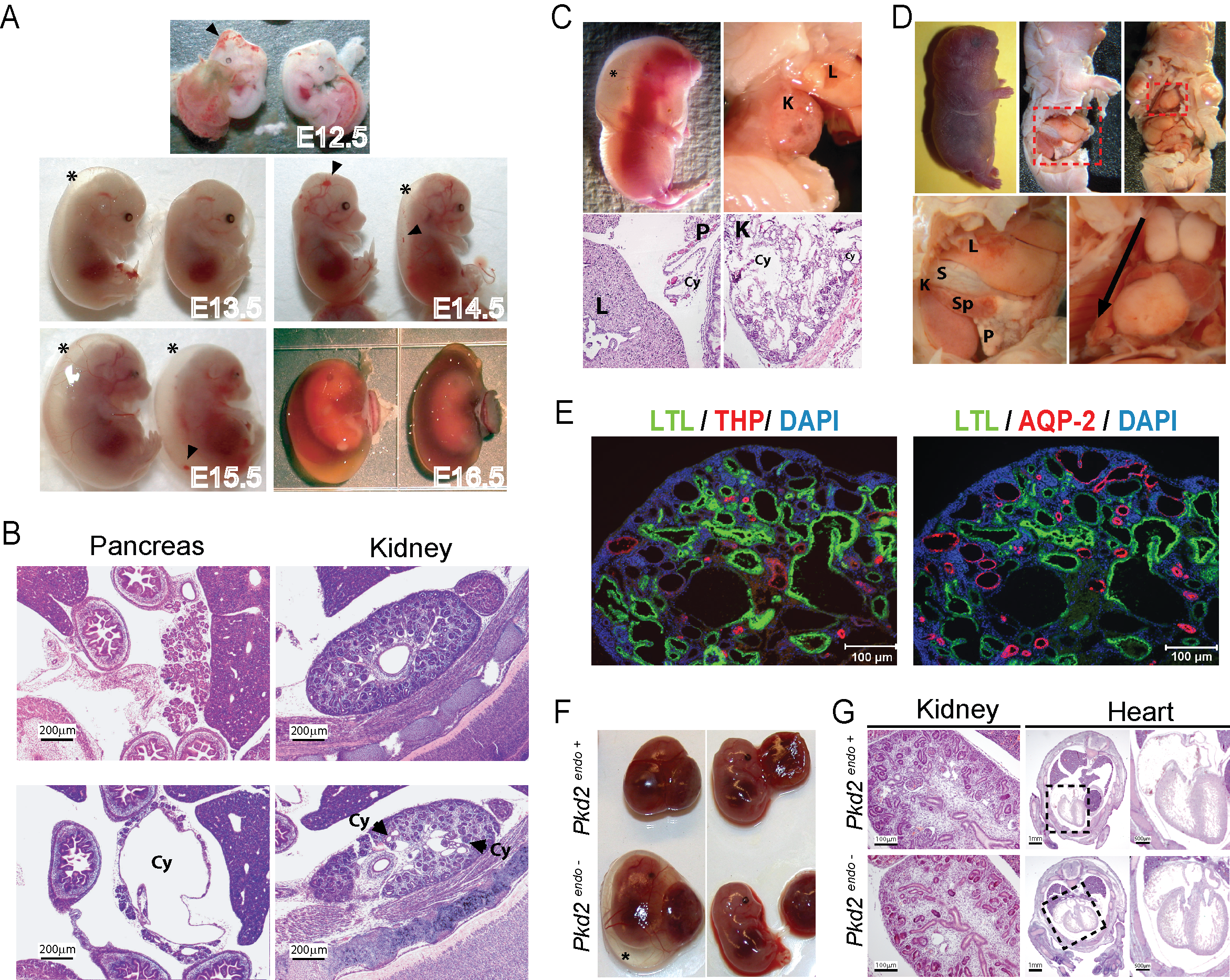

Supplement: Figure S2 — Phenotypes of Floxed and Mutant alleles of Pkd2. A. Pkd2Δ11-13/+ heterozygotes were bred and pregnancies harvested at various time points as indicated. The gross phenotypes of Pkd2Δ11-13/Δ11-13 embryos are demonstrated. The asterisk, “*”, indicates edema, Arrowheads denote areas of hemorrhage. The E16.5 embryos were dead and in the process of being resorbed. B. Hematoxylin and eosin staining of kidney and pancreas sections of normal (top) and mutant (bottom) E15.5 embryos. C. Pkd2cond/Δ11-13; Meox2-Cre+ mice were generated using standard breeding protcols. The top left panel shows the general appearance of a P0 Pkd2cond/Δ11-13; Meox2-Cre+ neonate with edema (asterisk). On the right, necropsy shows a cystic kidney. In the bottom panels, histopathology confirms the presence of cysts in the kidney (K) and pancreas (P). “Cy” indicates cysts. D. The top left panel shows the general appearance of another P0 Pkd2cond/Δ11-13; Meox2-Cre+ neonate. Necropsy of the pup, which died shortly after birth, demonstrates situs inversus (top middle and right panels). The areas in the red squares are magnified in the lower panels. Right-sided stomach and spleen (left lower panel) as well as dextrocardia (right lower panel, arrow) are seen. L: liver, P: pancreas, K: kidney, S: stomach, Sp: spleen, Cy: cyst. E. Cysts derive from all tubular segments. Cystic kidneys from the embryo in panel A stained with markers for proximal tubule (LTL, green), thick ascending limb (Tamm Horsfall, red) collecting duct (aquaporin-2, red) and Nuclei (DAPI, blue). F. Gross appearance of an E14.5 Pkd2endo− embryo and littermate control. The Pkd2endo− embryo has polyhydramnios indicated by the asterisk. At the right, the yolk sac was removed and neither embryo has edema. G. Haematoxylin-eosin staining of kidney and heart from an E13.5 Pkd2endo− embryo and littermate control. As in Pkd1endo−, embryos, there were no obvious histopathology abnormalities in the animals bearing the Tie-2 Cre recombinase (Pkd2endo− [file pone.0012821.s003.tif]
