# Supplementary material for: Pkd1 and Pkd2 Are Required for Normal Placental Development
Source: PLoS One. 2010 Sep 16;5(9):e12821. doi: 10.1371/journal.pone.0012821 (PMC2940908; doi:10.1371/journal.pone.0012821)
Supplement: Table S1 — Genotypes resulting from Pkd1+/− Intercrosses. (0.11 MB PDF) [file pone.0012821.s004.pdf]

Table S1: Genotypes resulting from *Pkd1*$^{+/-}$ Intercrosses.

Age (d.p.c.)	*Pkd1*$^{+/+}$	*Pkd1*$^{+/-}$	*Pkd1*$^{-/-}$
9.5	4	10	5
10.5	8	12	7
11.5	24	39	17
12.5	30	60	15
13.5	20	33	11
14.5	19	32	10
15.5	28	49	15
16.5	24	45	4
17.5	13	30	3 (2 dead)
18.5	9	15	0
