# Supplementary material for: Pkd1 and Pkd2 Are Required for Normal Placental Development
Source: PLoS One. 2010 Sep 16;5(9):e12821. doi: 10.1371/journal.pone.0012821 (PMC2940908; doi:10.1371/journal.pone.0012821)
Supplement: Table S2 — Genotypes resulting from mating of Pkd2 allele. (0.15 MB PDF) [file pone.0012821.s005.pdf]

Table S2: Genotypes resulting from mating of *Pkd2* allele.

A.

Age	*Pkd2*$^{+/+}$ n (%)	*Pkd2*$^{flox11\text{-}13/+}$ n(%)	*Pkd2*$^{flox11\text{-}13/flox11\text{-}13}$ n(%)	total
E13.5-E16.5	13 (25%)	28 (54%)	11 (21%)	52
E17.5-E19.5	14 (28.5%)	31 (63%)	13 (26%)	58
After Birth	31 (32%)	56 (58%)	7 (7%)	94

B.

Age	*Pkd2*$^{+/+}$ n (%)	*Pkd2*$^{\Delta Neo/+}$ n(%)	*Pkd2*$^{\Delta Neo / \Delta Neo}$ n(%)	total
E13.5-E16.5	7 (23%)	15 (50%)	8 (27%)	30
E17.5-E19.5	8 (25%)	15 (46%)	9 (28%)	32
After Birth	59 (23%)	133 (52%)	62 (24%)	254

C.

Age	*Pkd2*$^{+/+}$ n (%)	*Pkd2*$^{\Delta 11\text{-}13/+}$ n(%)	*Pkd2*$^{\Delta 11\text{-}13/\ \Delta 11\text{-}13}$ n(%)	total
E10.5-E12.5	8 (24%)	18 (55%)	7 (21%)	33
E13.5-E14.5	15 (21%)	39 (54%)	18 (25%)	72
E15.5-E16.5	35 (28%)	67 (54%)	22 (18%)	124
E17.5-E19.5	18 (31%)	37 (63%)	3 (5%)	58
After Birth	49 (32%)	104 (68%)	0 (0%)	153
